# Synergetic Effect of Li-Ion Concentration and Triple Doping on Ionic Conductivity of Li_7_La_3_Zr_2_O_12_ Solid Electrolyte

**DOI:** 10.3390/nano12172946

**Published:** 2022-08-26

**Authors:** Minh Hai Nguyen, Sangbaek Park

**Affiliations:** Department of Materials Science and Engineering, Chungnam National University, Daejeon 34134, Korea

**Keywords:** solid electrolytes, LLZO, Li-ion concentration, triple doping, Li-ion conductivity

## Abstract

Li_7_La_3_Zr_2_O_12_ (LLZO) is a promising and safe solid electrolyte for all-solid-state batteries. To achieve high ionic conductivity of LLZO, stabilizing the cubic phase and reducing Li loss during the sintering process is essential. Therefore, reducing the sintering temperature, which increases the sintering time for high-density pellets, is necessary. Herein, we investigate the change in the crystal structure, morphology, and Li ionic conductivity of LLZO pellets by triple doping with Al, Ga, and Ta and modulating the variation in initial Li concentrations. Interestingly, the proportion of the conductive cubic phase increased with increasing Li stoichiometry by 1.1 times, and this tendency was further accelerated by triple doping. The synergetic effects of triple doping and Li concentration also minimized Li loss during sintering. Accordingly, it provided a high-quality LLZO pellet with good ionic conductivity (3.6 × 10^−4^ S cm^−1^) and high relative density (97.8%). Notably, the LLZO pellet was obtained using a very short sintering process (40 min). Considering that the most time-consuming step is the sintering process for LLZO, this study can provide guidelines for the fast production and commercialization of LLZO electrolytes with high ionic conductivity.

## 1. Introduction

Lithium-ion batteries (LIBs) have been commercialized and extensively applied in various energy storage systems such as electric transportation and portable electronic devices [1,2,3]. LIBs with Li-rich liquid electrolytes exhibit high lithium-ion conductivities. However, a few serious safety issues can occur during charging and discharging, such as uncontrolled exothermic reactions, self-ignition, or chemical leakage due to excessive charging and internal short circuits [4,5]. Currently, all-solid-state Li-ion batteries (ASSLIBs) containing solid electrolytes are promising candidates to replace conventional Li-ion batteries in terms of extensive applications in different power sources with low risk and high reliability. Compared to LIBs, ASSLIBs exhibit outstanding properties such as higher safety, higher power density, lower chemical leakage, longer cycle life, and lower self-charging rate [1,6].

To date, extensive studies have been carried out to fabricate and develop various types of solid-state electrolytes with relatively high Li ionic conductivities [7,8]. Among them, Li garnet-type Li_7_La_3_Zr_2_O_12_ (LLZO) has been widely studied owing to several advantages such as high ionic conductivity, good thermal stability, excellent chemical stability against Li metals, and a wide range of operating temperatures and voltages [9,10]. LLZO contains two stable forms: cubic and tetragonal phases; the cubic phase exhibits a higher ionic conductivity (~10^−4^ S cm^−1^ at room temperature) when compared to tetragonal LLZO (~10^−6^ S cm^−1^ at room temperature) [11]. Therefore, fabricating LLZO with a high percentage of cubic phase is essential for high-performance ASSLIBs. To obtain LLZO with a highly conductive cubic structure, several studies have attempted to prolong the sintering time (typically more than 24 h) in the temperature range of 1000–1200 °C [12,13,14,15]. For the optimal performance of LLZO, extensive investigation related to Li-site and Zr-site doping has been carried out in few trivalent cations (Ga^3+^, Al^3+^) [16,17] and supervalent cations (Ta^5+^, Bi^5+^, Nb^5+^, Sb^5+^) [18,19,20,21], respectively, to stabilize the high-conductive phase (cubic phase) and increase the Li vacancy concentration, thereby enhancing the Li-ion conductivity of LLZO. Each dopant plays a specific role in the modification of the characteristics of LLZO by stabilizing the cubic phase. For example, Al-doping on Li^+^ sites stabilizes the cubic phase by increasing the Li vacancies in the crystal structure, and Al addition acts as a sintering aid that can improve the density of pellets [22,23]. Ga doping has a similar effect to Al substitution; however, it can stabilize the cubic phase of LLZO at a low sintering temperature of approximately 1000 °C [24], and some previous studies indicated that Ga-doped LLZO shows relatively high Li ionic conductivity when compared with other doping elements at Li sites [17,25]. In addition, Ta substitution on Zr sites stabilized the highly conductive cubic phase [18]; further, it will not impede Li-ion migration like Al-doping [18], and Ta is stable relative to Li [26]. However, systematic investigations of the effect of multiple doping with respect to the phase content or ionic conductivity of LLZO are still rare and elusive. In addition, during long-term sintering processes, Li loss occurs significantly, leading to the formation of secondary phases such as La_2_Zr_2_O_7_, which reduces the ionic conductivity of LLZO [27,28]. To avoid the loss of Li during preparation, the initial concentration of Li needs to be carefully controlled, and a short sintering process with an appropriate temperature should be considered. 

In this study, we synthesized solid-state LLZO electrolytes with triple doping (Al/Ga/Ta) and different initial Li^+^ concentrations in a short sintering time. The synergetic effects of triple doping and Li^+^ content on the crystal structure and Li-ion conductivity of LLZO were investigated and compared to those of Al-doped (mono-doped) LLZO. The results showed a phase transition and a change in the density of the pellets at different Li contents. Further, effective Li substitution and reduced Li loss were observed in the pellets prepared with triple doping at the optimized Li concentration after calcination and sintering, respectively. This demonstrates the importance of both the Li concentration and triple doping in the fabrication of highly ionic conductive LLZO electrolytes. Based on this synergetic effect, high-quality LLZO pellets were obtained, with the best ionic conductivity of 3.6 × 10^−4^ S cm^−1^ and a high relative density (97.8%). In addition, it is noteworthy that we calculated the percentage of cubic phase in the crystal structure of LLZO and examined the purity of the crystal phase based on the XRD Rietveld refinement method. This approach cannot be provided specifically through only XRD patterns, which have rarely been investigated in previous literature. Thus, our contribution to the LLZO field is not only control of the fabrication process but also tailoring the phase component of final products more efficiently, which will reduce the cost and energy for researching and manufacturing. We believe that our investigation can contribute to future studies related to LLZO electrolytes with high ionic conductivity and a short sintering process.

## 2. Materials and Methods

### 2.1. Preparation of Solid Electrolytes

Li_2_CO_3_ (KOJUNDO, 99.99%), La_2_O_3_ (KOJUNDO, 99.9%), ZrO_2_ (KOJUNDO, 98%), Al_2_O_3_ nanoparticles (Sigma-Aldrich, <50 nm particle size), Ga_2_O_3_ (KOJUNDO, 99.99%), and Ta_2_O_5_ (KOJUNDO, 99.9%) were used for the preparation of solid electrolytes. Owing to the hygroscopic nature of La_2_O_3_, it was heated at 900 °C for 12 h before grinding to remove absorbed moisture. The raw materials were weighed with a Li:La:Zr molar ratio of x:3:2 with x values 6.9, 7.7, and 8.4, which is assigned to 10.4%, 23.2%, and 34.4% excess Li, respectively. For the Al-doped LLZO, a specific amount of Al_2_O_3_ powder was added to achieve 0.25 mol of Al in the one-unit formula of LLZO samples with different Li doping levels (Al_6.9, Al_7.7, and Al_8.4), and the theoretical chemical formula was Li_x−0.75_Al_0.25_La_3_Zr_2_O_12_. To prepare triple-doped LLZO, Al_2_O_3_, Ga_2_O_3_, and Ta_2_O_5_ were added to obtain samples with the chemical formula Li_x−0.75_Al_0.172_Ga_0.072_La_3_Zr_1.982_Ta_0.018_O_12_, and these samples were denoted based on the initial Li^+^ concentration (AGT_6.9, AGT_7.7, and AGT_8.4). All precursor materials were mixed using planetary ball milling in ethanol (99.9%) at 250 rpm for 6 h. After drying the mixtures, the powder samples were collected and calcined at 900 °C for 6 h to obtain the early phase of LLZO. The calcined powders were reground at different rotational speeds (200, 300, 400, and 500 rpm) for 2 h and pressed into pellets at 200 MPa. The obtained pellets were covered with the same mother powder in MgO crucibles and sintered at 1250 °C for 40 min. Finally, all the pellets were polished and stored in a glove box.

### 2.2. Characterization

The phase compositions of all powder and pellet samples were analyzed by X-ray diffraction (XRD) analysis using a D8 ADVANCE instrument (BRUKER, Karlsruhe, Germany) with a Cu Kα radiation source (40 kV and 40 mA). To refine the crystalline structure, the Rietveld method was applied using a High Score Plus computer program (Malvern Panalytical Ltd., Malvern, UK). The morphological characteristics of the samples were analyzed using a field-emission scanning electron microscopy (FE-SEM) system (HITACHI S-4800, HITACHI, Tokyo, Japan). The densities of the pellet samples were measured using the Archimedes method with water. Inductively coupled plasma atomic emission spectroscopy (ICP-AES, Avio500, Perkin-Elmer, Waltham, MA, USA) was used to analyze the elemental compositions of the samples. The average particle size of the powder samples was measured using a laser scattering particle size analyzer (PSA, Helos KFS-MAGIC, Sympatec GmbH, Clausthal-Zellerfeld, Germany). The ionic conductivities of all pellets were determined using an impedance spectrometer (IVIUM potentiostat/galvanostat, IVIUM technologies, Eindhoven, Netherlands) in the frequency range of 1 Hz to 10 MHz. Two mirror-polished sides of all pellets were coated with silver paste (resistivity: ~10^−4^ Ω·cm) and subsequently connected to an impedance spectrometer via electrical wires. Conductivity measurements were performed at various temperatures (25–80 °C).

## 3. Results and Discussion

First, the effect of the second ball milling condition on the structural and morphological properties of the mother powders was investigated to optimize the quality of these powders for the preparation of pellets. Two types of doped LLZO with an initial Li^+^ concentration of 6.9 mol were used for this optimization. Figure S1 shows the XRD patterns of both the Al-doped and Al/Ga/Ta-doped LLZO powders after the second ball milling process (Table S1) at different rotation speeds. The powders without the second ball milling process, Al0, and AGT0, mainly displayed typical peaks of the cubic phase, which indicates that this phase was dominant in both the samples. This was also confirmed by the XRD Rietveld refinement results in Figure S2a,b with 73.7% and 72.9% of the cubic phase in Al0 and AGT0, respectively. The small peaks at 2θ values of 28.6° and 33.3° correspond to the presence of La_2_Zr_2_O_7_ (a secondary phase) in the crystal structure, which can be attributed to the insufficient Li source and Li loss during fabrication. The XRD Rietveld refinement results also revealed a reduction in the cubic phase and an improvement in the tetragonal phase when the rotation speed of the second ball milling process was increased (Figure S2). This indicates that the grinding process with high energy significantly affects the crystal structure of the mother powders. To obtain cubic LLZZO pellets from the mother powders with a low concentration of cubic phase, higher energy (high temperature and/or longer time) is required for the sintering process to increase the Li loss. Further, the sample without the second ball milling process showed a much larger particle size compared to the samples after the second grinding, while there was no significant difference in particle size between the ground powders (Table S1, Figure S3). For pellets prepared using large-sized particles, sintering at lower energy would not be sufficient to entirely sinter the particles, whereas sintering the pellet at higher energy results in porous grain boundaries between large grains, which can be favorable for the growth of lithium dendrites, leading to short-circuiting of the cell [29]. Therefore, the second ball milling process with a low rotation speed (200 rpm) was selected to optimize the quality of the mother powder for the preparation of pellets in this study.

**Figure 1:**
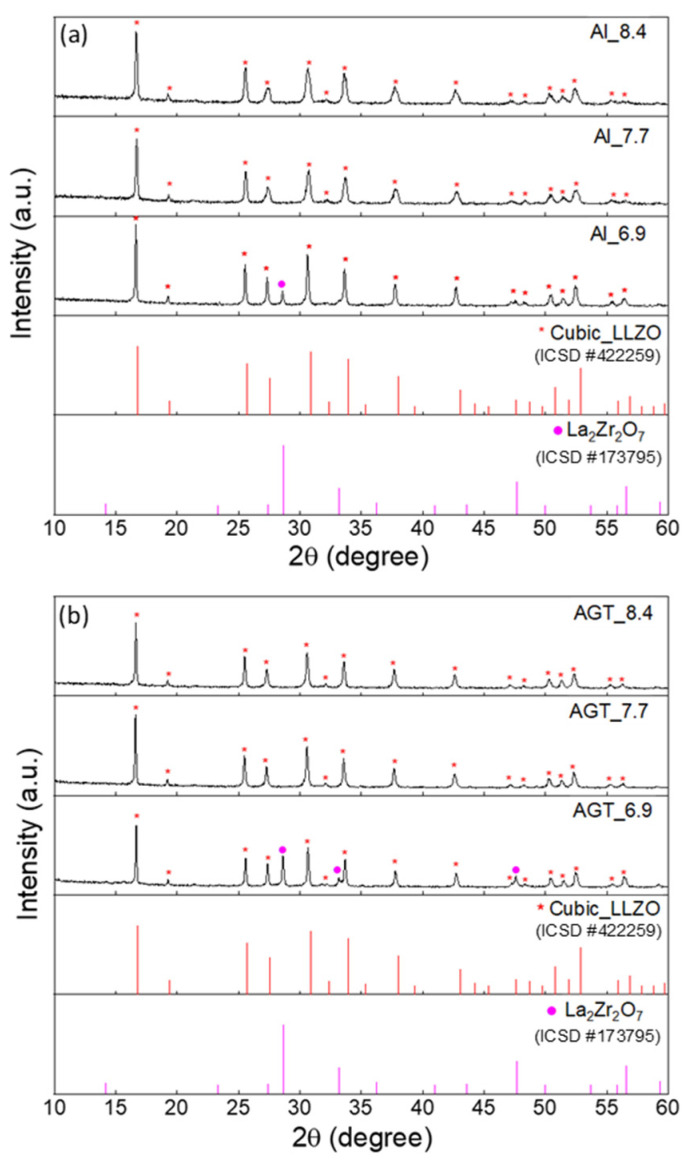
XRD patterns of (**a**) Al-doped LLZO powders and (**b**) Al/Ga/Ta-doped LLZO powders with different Li^+^ concentrations (6.6, 7.7, and 8.4 mol). The peaks marked by red and purple dots are attributed to cubic LLZO and La_2_Zr_2_O_7_, respectively. The red and purple vertical lines represent the standard diffraction peaks of cubic LLZO and La_2_Zr_2_O_7_, respectively.

The XRD patterns of the Al-doped LLZO powders and Al/Ga/Ta-doped LLZO powders with different Li^+^ concentrations after the second ball milling process under optimized conditions (200 rpm, 2 h) are shown in Figure 1. At a low Li content (x = 6.9), the cubic phase is present in both Al-doped and Al/Ga/Ta-doped LLZO samples along with a secondary phase (La_2_Zr_2_O_7_) (additional peaks at 28.6° and 33.3°). Using XRD Rietveld refinement, the major concentrations of the cubic phase in the Al_6.9 and AGT_6.9 powder samples were determined to be 74% and 64.1%, respectively (Figure S4a,b). The presence of the La_2_Zr_2_O_7_ phase in the structure of these samples can be attributed to Li loss and insufficient Li sources to form cubic LLZO at this initial Li concentration. Duvel et al. reported that Al^3+^ ions could occupy non-Li cation sites with a high Al content (above 0.2 mol per LLZO formula unit) [30]. Therefore, Al/Ga/Ta triple doping with a small concentration of each element may substitute Li^+^ sites more efficiently than Al doping with a high content (0.25 mol), resulting in a higher level of Li replacement in the AGT samples after thermal processes at high temperatures. This was also confirmed by the ICP-AES results with the difference in Li content between the Al_6.9 and AGT_6.9 samples (Table 1). As the initial Li concentration increases (x = 7.7 or 8.4), the cubic phase becomes dominant without the presence of a secondary phase in both the Al-doped and AGT-doped LLZO powders (Figure S4c–f). This indicates that a Li content of 7.7 mol or higher is sufficient for cubic LLZO formation with high purity. 

**Figure 2:**
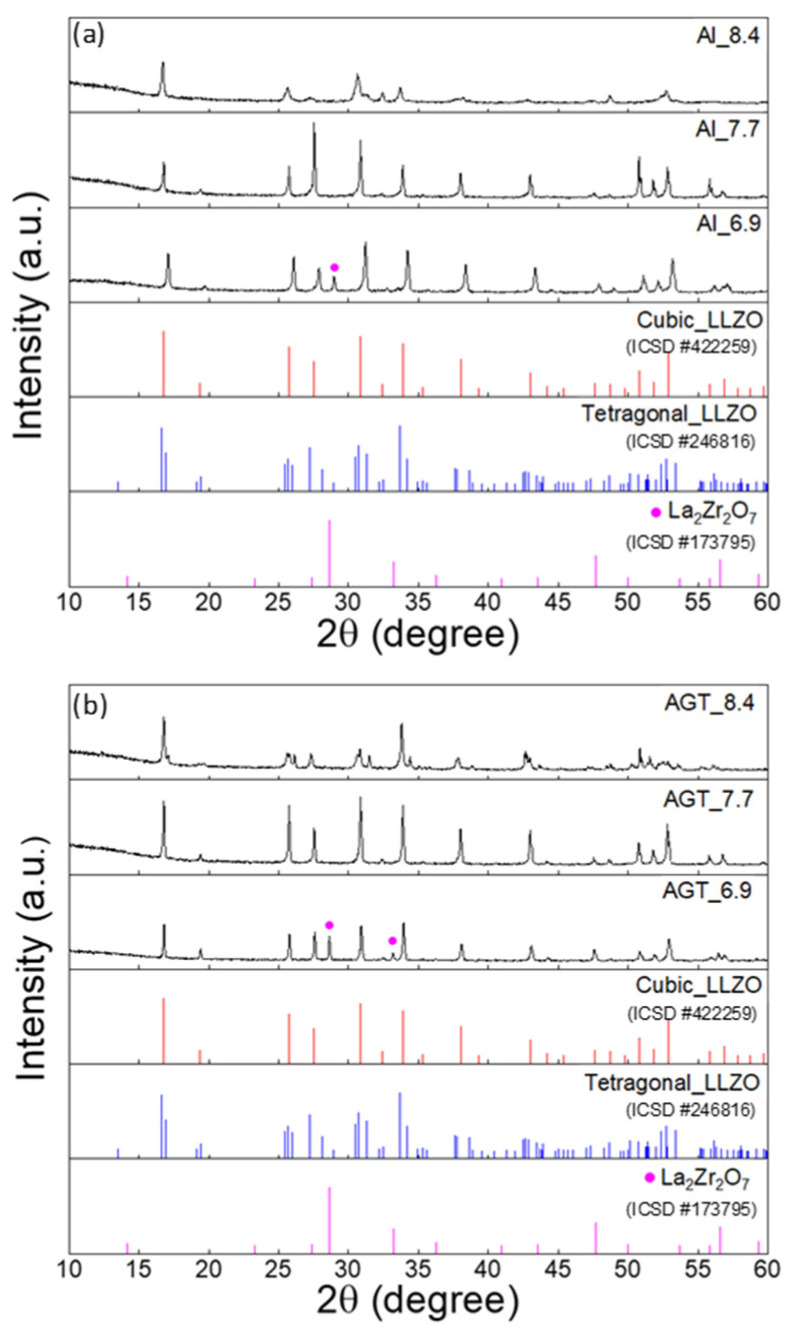
XRD patterns of (**a**) Al-doped LLZO pellets and (**b**) Al/Ga/Ta-doped LLZO pellets with different Li^+^ concentrations (6.6, 7.7, and 8.4 mol). The peaks marked by purple dots are attributed to La_2_Zr_2_O_7_. The red, blue and purple vertical lines represent the standard diffraction peaks of cubic LLZO, tetragonal LLZO and La_2_Zr_2_O_7_, respectively.

Figure 2 shows the XRD patterns of the sintered Al-doped LLZO pellets and Al/Ga/Ta-doped LLZO pellets with different Li^+^ concentrations. After the sintering process, the Al_6.9 and AGT_6.9 pellet samples still contained a majority of the cubic phase, and some small peaks of the secondary phase (La_2_Zr_2_O_7_) were also observed (Figure S5a,b) because of the low concentration of Li sources and the loss of Li during sintering. At an initial Li content of 7.7 mol, only typical diffraction peaks of the cubic phase are present in both Al-doped and AGT-doped LLZO pellets (Figure 2). A phase transformation of the pellets from cubic to tetragonal LLZO is observed when the Li concentration increases from 7.7 to 8.4 (Figure S5c–f); however, all the mother powder samples corresponding to these Li contents have high quality with a dominance of the cubic phase. This could be due to the distortion of unit cells to accommodate the further filling of excess Li atoms in specific Li vacancy sites [31]. These results suggest that the initial Li concentration plays a critical role in the formation of the final cubic LLZO pellets, and the Li content should be optimized with lower and upper limits at which the formation of the secondary phase and the transformation of the cubic phase can occur, respectively. 

**Figure 3:**
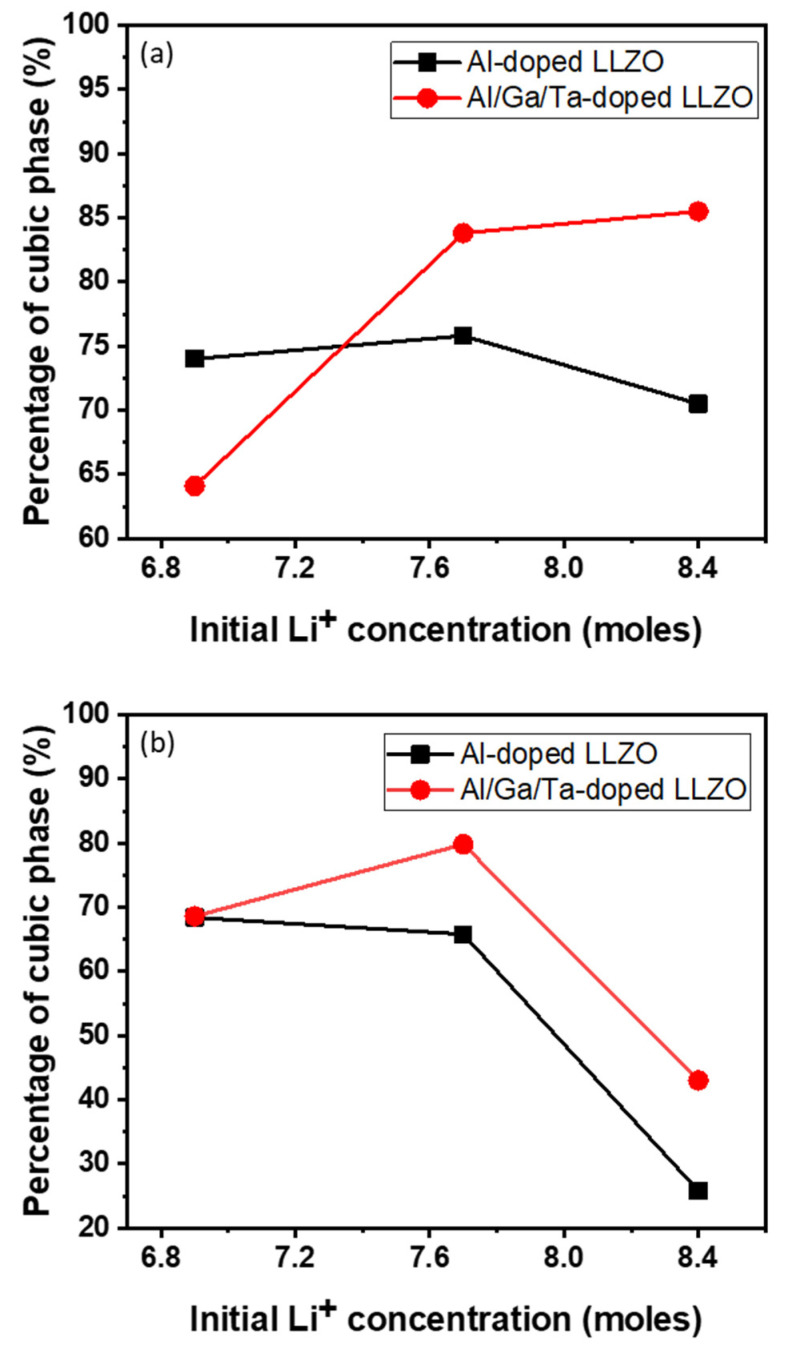
Relative weight fraction of cubic phase of (**a**) doped powders and (**b**) doped pellets by the XRD Rietveld refinement.

The phase compositions of all powder and pellet samples were analyzed using the XRD Rietveld refinement method, and the change in the concentration of the cubic phase in samples with two types of doping and different Li concentrations is shown in Figure 3. For the powder samples, although at low Li contents, the formation of a cubic phase in AGT-doped LLZO is lower because of the significant secondary phase, triple doped (Al/Ga/Ta) LLZO shows a relatively higher cubic phase content compared to mono doped LLZO (Al doping) when the initial Li concentration is increased to 7.7 mol or higher. In addition, AGT doping facilitated better cubic phase stability in LLZO than Al doping after sintering the pellets. The results of XRD Rietveld refinement analysis show that the percentage of cubic phase rather increased from 68% to more than 80% for Al/Ga/Ta-doped LLZO when Li excess amount increased from 10.4% to 23.2%, whereas the percentage of cubic phase decreased from 68% to 65% for Al-doped LLZO when Li excess amount increased from 10.4% to 23.2%. As a result, sample AGT_7.7 (23.2% excess Li) showed the highest percentage of cubic phase, which demonstrated the merit of optimized excess Li addition combined with triple doping (Figure 3), which enhances the formation and stability of the cubic phase in both the powder and pellet samples.

**Figure 4:**
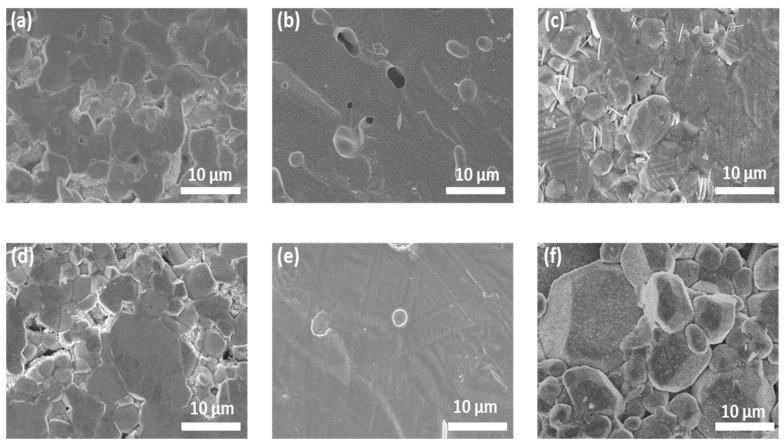
SEM images of the cross-sections of sintered pellets: (**a**) Al_6.9, (**b**) Al_7.7, (**c**) Al_8.4, (**d**) AGT_6.9, (**e**) AGT_7.7, and (**f**) AGT_8.4.

The cross-sectional morphology of the sintered pellets is shown in Figure 4, and digital images of all pellets are shown in Figure S6. At an optimized Li concentration (7.7 mol), both single- and triple-doped LLZO pellets show smooth surfaces with small closed pores (Figure 4b,e), while many more grain boundaries are observed in other samples with lower and higher Li contents. Further, the Al_7.7 and AGT_8.4 pellets exhibited a light-yellow color (Figure S6), which is usually observed from the good sintering conditions of ceramics [32]. This indicates that the Al_7.7 and AGT_7.7 samples were well sintered with high density, which can result in low grain boundary (GB) resistance, thereby improving the ionic conductivity of these pellets [14]. By comparison, a large number of grain boundaries in the samples with lower and higher Li contents contribute to lower ionic conductivity. Therefore, a suitable initial Li concentration is important to ensure good sintering of the LLZO pellets.

**Figure 5:**
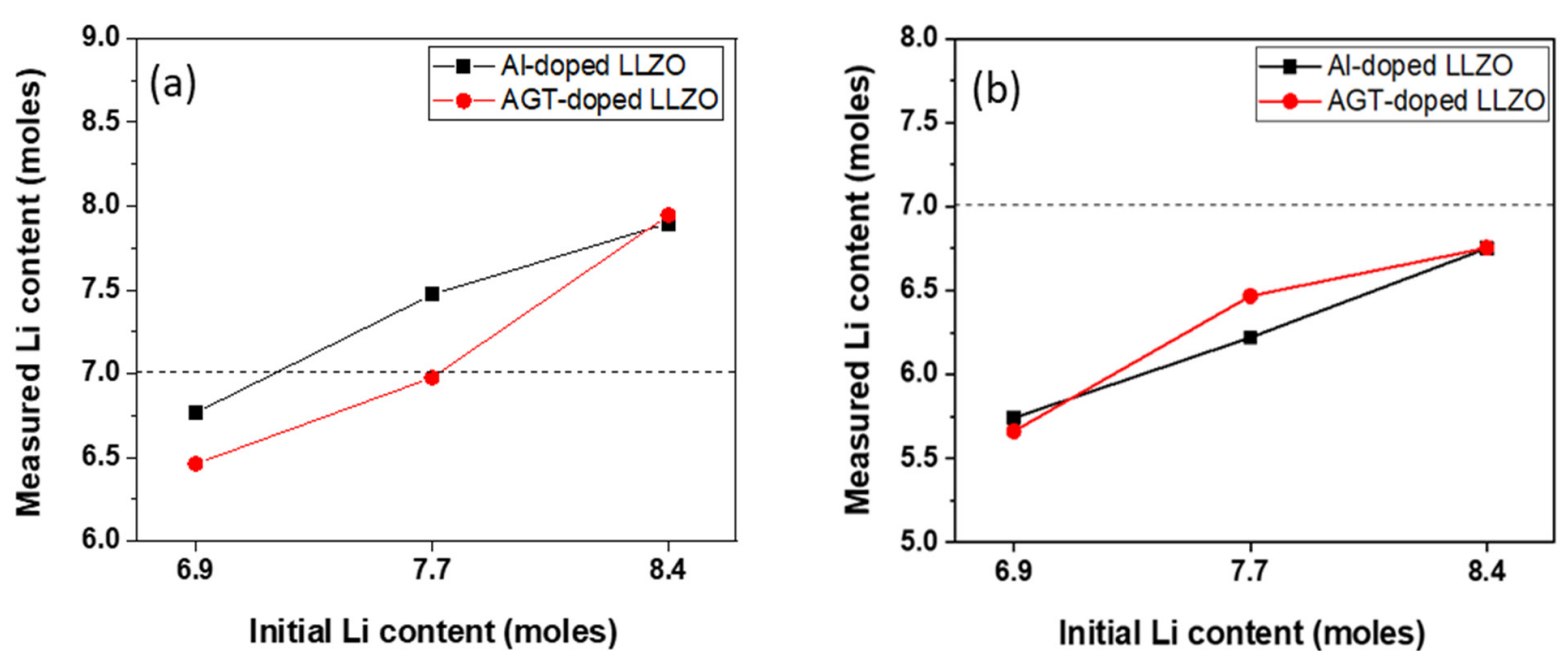
Change in Li-ion concentration of (**a**) calcined powders and (**b**) sintered pellets.

Figure 5 and Table 1 show the Li contents of the Al-doped and AGT-doped LLZO powders and sintered pellets, which were analyzed by ICP-AES measurements. All powders exhibit a slight reduction in Li content after calcination at 900 °C for 6 h. Compared to the mono-doped LLZO powders with initial Li contents of 6.9 and 7.7 mol, the lower Li concentration observed in triple-doped LLZO powders could be attributed to the more effective replacement of Li sites by appropriate amounts of Al, Ga, and Ta dopants rather than a high content of Al dopant alone [30]. This indicates efficient doping on the Li site of the LLZO structure after calcination by introducing three elements (Al, Ga, and Ta) in the powders prepared with the aforementioned Li concentrations. In the powders with high Li content (x = 8.4), the continuous filling of Li in the LLZO structure from a large Li source could impede the doping on Li sites of other elements, leading to no significant difference in the measured Li content between the Al-doped and AGT-doped powders. After sintering, all Li contents in the pellets were further decreased, with values lower than 7 mol. This demonstrates the presence of Li vacancies in all sintered pellets, which is favorable for Li movement. Remarkably, the AGT_7.7 pellet shows the lowest Li loss compared to the other samples, which can be attributed to the effective sintering with fewer grain boundaries and small closed pores. Based on the results, the synergetic effect of triple doping and optimized Li content can significantly reduce Li reduction during sintering, which is ideal for high-temperature and long-duration processes.

**Figure 6:**
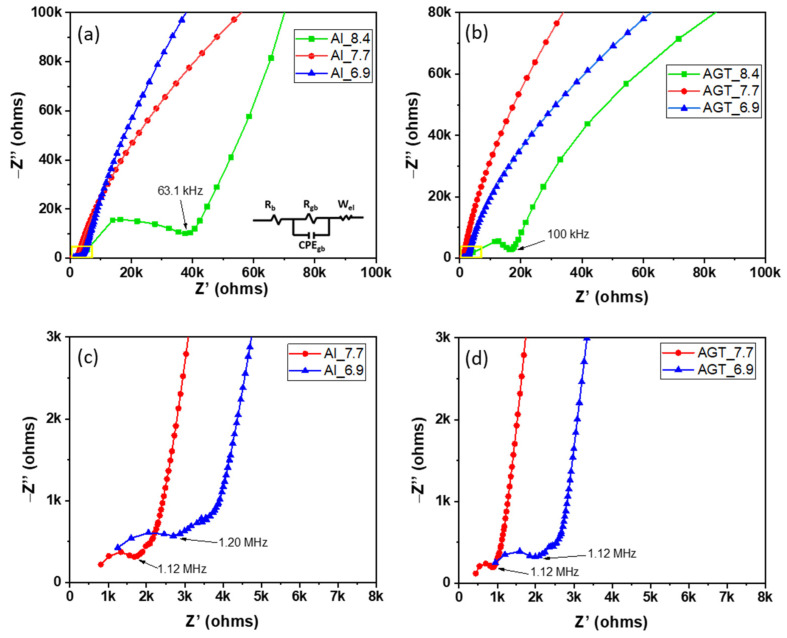
Nyquist plots of (**a**,**c**) the Al-doped LLZO pellets and (**b**,**d**) Al/Ga/Ta-doped LLZO pellets with different Li^+^ concentrations (6.6, 7.7, and 8.4 mol). The fitting circuit is provided in the inset.

**Figure 7:**
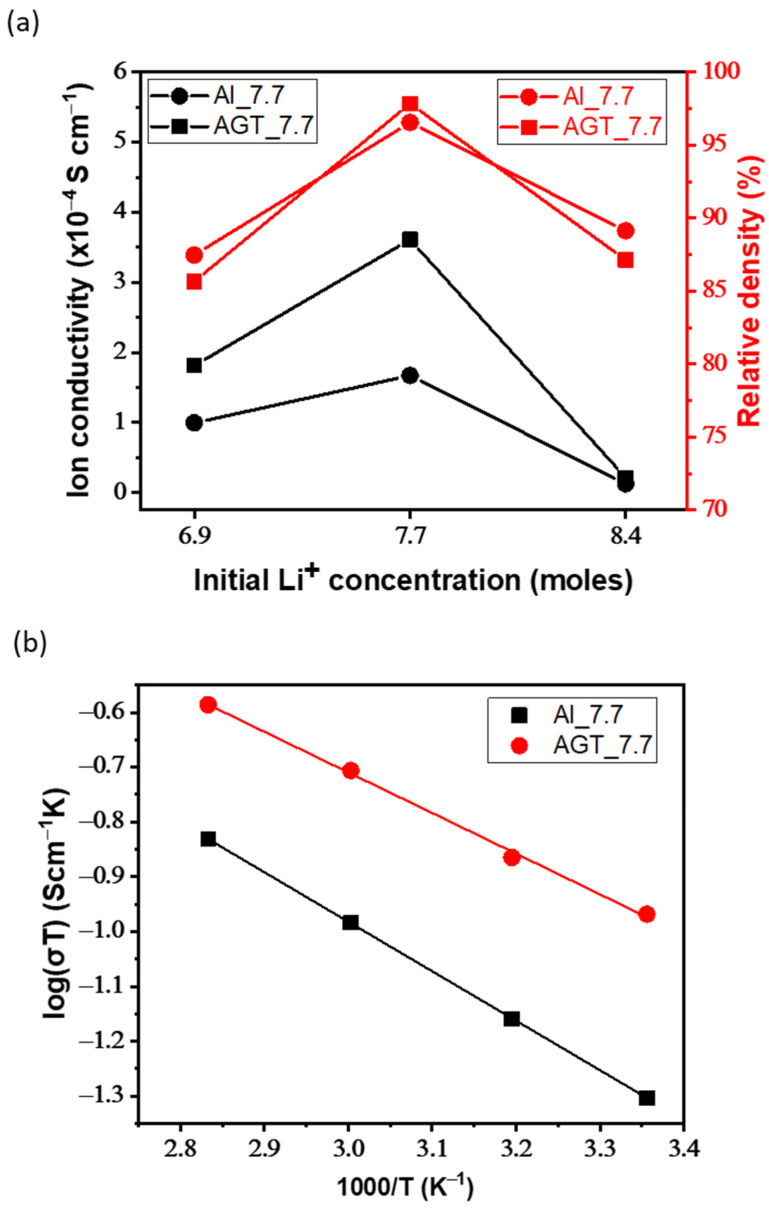
(**a**) Total ionic conductivity and relative density of pellets with different initial Li^+^ concentrations (6.6, 7.7, and 8.4 mol) at 25 °C. (**b**) Arrhenius plots of Al_7.7 and AGT_7.7 samples.

Figure 6a–d shows the EIS curves of the sintered Al- and AGT-doped LLZO pellets with different initial Li contents. An equivalent circuit model (R_b_ (R_gb_//CPE_gb_) W_el_) was also presented, where R_b_, R_gb_, CPE_gb_, and W_el_ are the bulk resistance, GB resistance, constant phase element, and Warburg diffusion element, respectively. In Figure 6a,b, Al_8.4 and AGT_8.4 show large GB impedance semi-circles with terminal frequencies of approximately 63.1 and 100 kHz, respectively, while the other curves inside the yellow squares exhibit much lower diameters. In the high-frequency view of Nyquist plots (Figure 6c), the Al_7.7 sample shows a smaller curve than Al_6.9, both semi-circle curves correspond to the GB resistance with terminal frequencies of 1.12 and 1.2 MHz, respectively, and diffusion tails in medium and low frequencies are assigned to the Warburg impedances. Similar curves are observed in the case of AGT_7.7 and AGT_6.9 pellets; however, their semi-circles at higher frequencies have smaller diameters than those of Al_7.7 and Al_6.9 samples. The conductivities and relative densities of the pellets are presented in Table 2 and Figure 7a. For each doping type, pellets with an initial Li concentration of 7.7 mol show the highest conductivity values due to the high concentration of the conductive phase (cubic LLZO) without any secondary phase. Although there is a high percentage of cubic phase in the structures, at low Li content (x = 6.9), the samples exhibit lower Li-ion conductivities than the samples with optimized Li concentration (x = 7.7) because of the presence of the La_2_Zr_2_O_7_ phase. When the Li content is increased to 8.4 mol, the tetragonal phase becomes dominant in the crystal structure of the pellets owing to the phase transformation, leading to a significant decrease in ionic conductivities. In particular, the AGT_7.7 sample showed the best ionic conductivity (3.6 × 10^−4^ S cm^−1^), while the conductivity of the Al_7.7 sample was approximately 1.7 × 10^−4^ S cm^−1^. In addition, at the same Li concentration, other triple-doped (AGT-doped) samples showed better Li-ion conductivities than the mono-doped (Al-doped) samples. The enhanced Li-ion conductivity of LLZO with triple doping could be attributed to the positive effect of each additional doping element (Ga, Ta) on the Al-doped LLZO. Ga reduced the hindrance of Li-ion mobility due to the lower occupation percentage on Li1 sites (24 d Li sites) than Al and also enlarged the lattice for Li-ion transport because of its large size [33]. Further, the additional Ta doping can move Al from 24 d to 96 h Li sites (Li2 sites), thereby providing more pathways and Li vacancies for Li-ion movement [34]. Investigation of LLZO with other single and dual doping such as Ga-doped LLZO (Ga_7.7) and Al/Ga-doped LLZO (AG_7.7) with Li^+^ concentration of 7.7 mol was also carried out for comparison (Figure S8 and Table S2), they show lower ionic conductivity (2.0 × 10^−4^ and 3.2 × 10^−4^ S cm^−1^, respectively) compared to sample AGT_7.7. It points out that all doping elements play important roles in the improvement of Li-ion conductivity of LLZO. The relative densities of all the pellets are presented in Table 2 and Figure 7a. Low relative density values were observed for the samples prepared with a low Li concentration (x = 6.9). Further increasing the Li content to 7.7 mol leads to a significant improvement in the relative density. The relative densities of Al_7.7 and AGT_7.7 samples reached 96.55% and 97.84%, respectively. Interestingly, the relative density begins to decrease with increasing Li content (x = 8.4), which is also confirmed by the SEM images of the sintered pellets in Figure 4c,f. This can be explained by the fact that at a high initial Li content, more Li tends to be located at the GB regions, and they can be easily evaporated in the form of Li_2_O vapors, consequently leaving gaps in the samples and reducing the density. This indicates the important role of the Li content in the formation of cubic LLZO and also the relative density of the pellets. The temperature dependence of the ionic conductivity of both the Al-doped and AGT-doped samples is displayed as Arrhenius plots in Figure 7b, and the EIS results of these samples at different temperatures are shown in Figure S7. The linear shape of the plots indicates that there was no change in the structure and components of the pellets during the measurement in the temperature range of 25–80 °C. At all temperatures, triple-doped LLZO shows a higher Li ionic conductivity than Al-doped LLZO. Further, the activation energy of triple-doped LLZO (0.34 eV) was lower than that of Al-doped LLZO (0.41 eV). This demonstrates an advantage for Li ionic transport with low activation energy, which has potential for solid-state battery applications [35]. 

Regarding fast densification, there are several previous works applying different advanced methods to reduce the duration of the sintering process. The list of fast-sintered LLZO pellets is summarized in Table S3, indicating that most previous works applied complex processes containing expensive equipment or consumables to compensate for the Li loss and the phase change during the fast densification. Ihrig et al. prepared Al/Ta-doped LLZO pellets by Ultrafast High-temperature Sintering (UHS) with an AC/DC power source; the sintering temperature reached 1500 °C, but the duration was only 10 s, and the best sample showed the ionic conductivity of 0.12 mS cm^−1^ at room temperature and the relative density of 93% [36]. Allen et al. applied the hot-pressing method with lower temperature (1050 °C) but longer time (1 h); the Li-ion conductivity and density of the pellet were 0.37 mS cm^−1^ and 98%, respectively [18]. The spark plasma sintering method was also applied to prepare LLZO pellet with relatively high ionic conductivity (0.69 mS cm^−1^) and density (95.5%) with low temperature (1000 °C) and short sintering time (10 min) [37]. Another study used high-quality Pt crucibles for the short sintering process (1250 °C, 40 min) to reduce the loss of Li, and the final pellet displayed high ionic conductivity (0.64 mS cm^−1^) and high density (95 %) [32]. In our study, we concentrated on the modification of the LLZO component by adding three different dopants (Al, Ga, and Ta) and controlling initial Li contents while applying a conventional fabrication process, which is cost-effective and easy to approach. We applied furnace sintering at 1250 °C for a short time (40 min), and the ionic conductivity of our best sample was 0.36 mS cm^−1^ (Table 2). This sample also has very high relative density (97.84%) (Table 2), and the cross-sectional SEM image of the pellet shows a dense surface with small closed pores and an almost negligible gap between each grain. Moreover, the pellet, after sintering, was stabilized with a high percentage of cubic phase in its structure (~80%) (Figure 3 and Figure S5). All the above results demonstrate efficient calcination with a short duration for good-quality LLZO pellets in this study. The ionic conductivity value of AGT_7.7 can be improved further by optimizing the ratio of doping elements and/or coarsening the grains. Actually, sample Al-doped LLZO with Li content of 7.7 mol was also prepared with a much longer sintering duration (24 h) for grain coarsening to reduce the grain boundary resistance term. As a result, the ionic conductivity of sample Al_7.7 was improved (0.33 mS cm^−1^) after extending the sintering time (Figure S9 and Table S3), revealing the decrease in grain boundary resistance. Remarkably, the ionic conductivity of Al_7.7 for 24 h sintering was still lower compared to that of sample AGT_7.7 (triple-doped LLZO with Li content of 7.7) for 40 min sintering. It indicates that triple doping plays an important role in the fabrication of high-quality LLZO pellets with a very short sintering time.

## 4. Conclusions

The synergetic effect of triple doping and Li concentration on the Li ionic conductivity of LLZO was investigated. Cubic LLZO was observed with a minority of secondary phase (La_2_Zr_2_O_7_) at a low initial Li content (x = 6.9). As the Li concentration increased to 7.7 mol, the cubic phase became dominant without the presence of a secondary phase. The phase transition from cubic to tetragonal LLZO occurred at high Li concentrations (8.4 mol). At each Li concentration, triple-doped LLZO with Al, Ga, and Ta dopants showed better ionic conductivity and a more stable cubic phase compared to single Al-doped LLZO. Based on the combination of these modifications, AGT_7.7 achieved the highest Li-ion conductivity (3.6 × 10^−4^ S cm^−1^) with a high relative density (97.8%). Notably, this high ionic conductivity of LLZO was obtained by a very short sintering process (duration = 40 min). We believe that this study can be a basis for future investigations related to the preparation of multiple-doped LLZO solid electrolytes with optimized Li concentrations for high-quality and low-energy consuming solid-state Li batteries. 

## Figures and Tables

**Table 1:** Composition of calcined powders and sintered pellets by ICP-AES.

Sample		Atomic Ratio				
		Li	Zr	Al	Ga	Ta
Calcined powder	Al_6.9	6.77	1.86	0.31	-	-
	Al_7.7	7.47	1.86	0.35	-	-
	Al_8.4	7.90	1.85	0.34	-	-
	AGT_6.9	6.46	1.83	0.21	0.07	0.02
	AGT_7.7	6.98	1.88	0.23	0.07	0.02
	AGT_8.4	7.95	1.85	0.23	0.07	0.02
Sintered pellet	Al_6.9	5.74	1.73	0.29	-	-
	Al_7.7	6.22	1.80	0.29	-	-
	Al_8.4	6.75	1.83	0.22	-	-
	AGT_6.9	5.66	1.77	0.22	0.08	0.02
	AGT_7.7	6.47	1.82	0.22	0.07	0.02
	AGT_8.4	6.75	1.79	0.19	0.06	0.01

**Table 2:** Summary of total (bulk + grain-boundary) ionic conductivity at 25 °C and relative density of pellets.

Sample	Al_6.9	Al_7.7	Al_8.4	AGT_6.9	AGT_7.7	AGT_8.4
Ion conductivity (×10^−4^ S cm^−1^)	1.0	1.7	0.1	1.8	3.6	0.2
Relative density (%)	87.47	96.55	89.14	85.65	97.84	87.14

## Data Availability

The data presented in this study are available on a reasonable request from the corresponding author.

## Supplementary Materials

The following supporting information can be downloaded at: https://www.mdpi.com/article/10.3390/nano12172946/s1, Figure S1: XRD patterns of (a) Al-doped LLZO powders and (b) Al/Ga/Ta-doped LLZO powders after the second ball milling process with different rotation speeds (0, 200, 300, 400, and 500 rpm); Figure S2: XRD Rietveld refinement results of Al-doped LLZO and AGT-doped LLZO powders prepared with Li content of 6.9 mol after the second ball milling process with different rotation speeds (0, 200, 300, 400 and 500 rpm): (a) Al0, (b) AGT0, (c) Al200, (d) AGT200, (e) Al300, (f) AGT300, (g) Al400, (h) AGT400, (i) Al500, and (j) AGT500; Figure S3: SEM images of the Al/Ga/Ta-doped LLZO powders after the second ball milling process at (a) 0 rpm, (b) 200 rpm, (c) 300 rpm, (d) 400 rpm, and (e) 500 rpm for 2 h; Figure S4: XRD Rietveld refinement results of powder samples after the second ball milling process at 200 rpm for 2 h: (a) Al_6.9, (b) AGT_6.9, (c) Al_7.7, (d) AGT_7.7, (e) Al_8.4, and (f) AGT_8.4; Figure S5: XRD Rietveld refinement results of pellets: (a) Al_6.9, (b) AGT_6.9, (c) Al_7.7, (d) AGT_7.7, (e) Al_8.4, and (f) AGT_8.4; Figure S6: Digital image of pellets; Figure S7: Nyquist plots of (a) Al_7.7 and (b) AGT_7.7 samples at 40–80 °C; Figure S8: Nyquist plots of Al-doped LLZO, Ga-doped LLZO, Al/Ga-doped LLZO, and Al/Ga/Ta-doped LLZO pellets with Li^+^ concentration of 7.7 mol; Figure S9: Nyquist plots of Al-doped LLZO prepared with Li^+^ concentration of 7.7 mol and sintering time of 24 h; Table S1: Average particle size of Al/Ga/Ta-doped LLZO powders prepared with different ball milling parameters through PSA measurement; Table S2: Summary of total ionic conductivity at 25 °C of Al-doped LLZO, Ga-doped LLZO, Al/Ga-doped LLZO, and Al/Ga/Ta-doped LLZO pellets with Li^+^ concentration of 7.7 mol. Table S3: Comparison of LLZO pellets prepared by fast sintering methods in this work and other studies. References in Table S3: [18,31,32,36,37,38].

## References

[1] Tarascon J.M., Armand M. (2001). Issues and Challenges Facing Rechargeable Lithium Batteries. Nature.

[2] Gwon H., Hong J., Kim H., Seo D.H., Jeon S., Kang K. (2014). Recent Progress on Flexible Lithium Rechargeable Batteries. Energy Environ. Sci..

[3] Bruce P.G., Freunberger S.A., Hardwick L.J., Tarascon J.M. (2011). Li–O_2_ and Li–S Batteries with High Energy Storage. Nat. Mater..

[4] Balakrishnan P.G., Ramesh R., Prem Kumar T. (2006). Safety Mechanisms in Lithium-Ion Batteries. J. Power Sources.

[5] Kil E.-H., Choi K.-H., Ha H.-J., Xu S., Rogers J.A., Kim M.R., Lee Y.-G., Kim K.M., Cho K.Y., Lee S.-Y. (2013). Imprintable, Bendable, and Shape-Conformable Polymer Electrolytes for Versatile-Shaped Lithium-Ion Batteries. Adv. Mater..

[6] Sun C., Liu J., Gong Y., Wilkinson D.P., Zhang J. (2017). Recent Advances in All-Solid-State Rechargeable Lithium Batteries. Nano Energy.

[7] Ji F., Xiao S., Cheng J., Li D., Liao J., Guo Y., Zhang H., Zhang S., Wei Y., Liu Y. (2022). Low-Cost and Facile Synthesis of LAGP Solid State Electrolyte via a Co-Precipitation Method. Appl. Phys. Lett..

[8] Cheng J., Hou G., Chen Q., Li D., Li K., Yuan Q., Wang J., Ci L. (2022). Sheet-like Garnet Structure Design for Upgrading PEO-Based Electrolyte. Chem. Eng. J..

[9] Zhu Y., He X., Mo Y. (2015). Origin of Outstanding Stability in the Lithium Solid Electrolyte Materials: Insights from Thermodynamic Analyses Based on First-Principles Calculations. ACS Appl. Mater. Interfaces.

[10] Cao C., Li B.Z., Wang X.L., Zhao X.B., Han W.Q. (2014). Recent Advances in Inorganic Solid Electrolytes for Lithium Batteries. Front. Energy Res..

[11] Awaka J., Kijima N., Hayakawa H., Akimoto J. (2009). Synthesis and Structure Analysis of Tetragonal Li_7_La_3_Zr_2_O_12_ with the Garnet-Related Type Structure. J. Solid State Chem..

[12] Murugan R., Thangadurai V., Weppner W. (2007). Fast Lithium Ion Conduction in Garnet-Type Li_7_La_3_Zr_2_O_12_. Angew. Chem. Int. Ed..

[13] Kotobuki M., Munakata H., Kanamura K., Sato Y., Yoshida T. (2010). Compatibility of Li_7_La_3_Zr_2_O_12_ Solid Electrolyte to All-Solid-State Battery Using Li Metal Anode. J. Electrochem. Soc..

[14] Kumazaki S., Iriyama Y., Kim K.H., Murugan R., Tanabe K., Yamamoto K., Hirayama T., Ogumi Z. (2011). High Lithium Ion Conductive Li_7_La_3_Zr_2_O_12_ by Inclusion of Both Al and Si. Electrochem. Commun..

[15] Ohta S., Seki J., Yagi Y., Kihira Y., Tani T., Asaoka T. (2014). Co-Sinterable Lithium Garnet-Type Oxide Electrolyte with Cathode for All-Solid-State Lithium Ion Battery. J. Power Sources.

[16] Buschmann H., Dölle J., Berendts S., Kuhn A., Bottke P., Wilkening M., Heitjans P., Senyshyn A., Ehrenberg H., Lotnyk A. (2011). Structure and Dynamics of the Fast Lithium Ion Conductor “Li_7_La_3_Zr_2_O_12_”. Phys. Chem. Chem. Phys..

[17] Bernuy-Lopez C., Manalastas W., Lopez Del Amo J.M., Aguadero A., Aguesse F., Kilner J.A. (2014). Atmosphere Controlled Processing of Ga-Substituted Garnets for High Li-Ion Conductivity Ceramics. Chem. Mater..

[18] Allen J.L., Wolfenstine J., Rangasamy E., Sakamoto J. (2012). Effect of Substitution (Ta, Al, Ga) on the Conductivity of Li_7_La_3_Zr_2_O_12_. J. Power Sources.

[19] Wagner R., Rettenwander D., Redhammer G.J., Tippelt G., Sabathi G., Musso M.E., Stanje B., Wilkening M., Suard E., Amthauer G. (2016). Synthesis, Crystal Structure, and Stability of Cubic Li_7-x_La_3_Zr_2-x_Bi_x_O_12_. Inorg. Chem..

[20] Ohta S., Kobayashi T., Asaoka T. (2011). High Lithium Ionic Conductivity in the Garnet-Type Oxide Li_7−X_ La_3_(Zr_2−X_, Nb_X_)O_12_ (X = 0–2). J. Power Sources.

[21] Ramakumar S., Satyanarayana L., Manorama S.V., Murugan R. (2013). Structure and Li+ Dynamics of Sb-Doped Li_7_La_3_Zr_2_O_12_ Fast Lithium Ion Conductors. Phys. Chem. Chem. Phys..

[22] Kotobuki M., Kanamura K., Sato Y., Yoshida T. (2011). Fabrication of All-Solid-State Lithium Battery with Lithium Metal Anode Using Al_2_O_3_-Added Li_7_La_3_Zr_2_O_12_ Solid Electrolyte. J. Power Sources.

[23] Jin Y., McGinn P.J. (2011). Al-Doped Li_7_La_3_Zr_2_O_12_ Synthesized by a Polymerized Complex Method. J. Power Sources.

[24] Song S., Yan B., Zheng F., Duong H.M., Lu L. (2014). Crystal Structure, Migration Mechanism and Electrochemical Performance of Cr-Stabilized Garnet. Solid State Ion..

[25] Jalem R., Rushton M.J.D., Manalastas W., Nakayama M., Kasuga T., Kilner J.A., Grimes R.W. (2015). Effects of Gallium Doping in Garnet-Type Li_7_La_3_Zr_2_O_12_ Solid Electrolytes. Chem. Mater..

[26] Li Y., Wang C.A., Xie H., Cheng J., Goodenough J.B. (2011). High Lithium Ion Conduction in Garnet-Type Li_6_La_3_ZrTaO_12_. Electrochem. Commun..

[27] Huang M., Liu T., Deng Y., Geng H., Shen Y., Lin Y., Nan C.W. (2011). Effect of Sintering Temperature on Structure and Ionic Conductivity of Li_7−x_La_3_Zr_2_O_12−0.5x_ (X = 0.5~0.7) Ceramics. Solid State Ion..

[28] Huang M., Dumon A., Nan C.W. (2012). Effect of Si, In and Ge Doping on High Ionic Conductivity of Li_7_La_3_Zr_2_O_12_. Electrochem. Commun..

[29] Ren Y., Shen Y., Lin Y., Nan C.-W. (2019). Microstructure Manipulation for Enhancing the Resistance of Garnet-Type Solid Electrolytes to “Short Circuit” by Li Metal Anodes. ACS Appl. Mater. Interfaces.

[30] Düvel A., Kuhn A., Robben L., Wilkening M., Heitjans P. (2012). Mechanosynthesis of Solid Electrolytes: Preparation, Characterization, and Li Ion Transport Properties of Garnet-Type Al-Doped Li_7_La_3_Zr_2_O_12_ Crystallizing with Cubic Symmetry. J. Phys. Chem. C.

[31] Rangasamy E., Wolfenstine J., Sakamoto J. (2012). The Role of Al and Li Concentration on the Formation of Cubic Garnet Solid Electrolyte of Nominal Composition Li_7_La_3_Zr_2_O_12_. Solid State Ion..

[32] Huang X., Lu Y., Guo H., Song Z., Xiu T., Badding M.E., Wen Z. (2018). None-Mother-Powder Method to Prepare Dense Li-Garnet Solid Electrolytes with High Critical Current Density. ACS Appl. Energy Mater..

[33] Wolfenstine J., Ratchford J., Rangasamy E., Sakamoto J., Allen J.L. (2012). Synthesis and High Li-Ion Conductivity of Ga-Stabilized Cubic Li_7_La_3_Zr_2_O_12_. Mater. Chem. Phys..

[34] Shin D.O., Oh K., Kim K.M., Park K.Y., Lee B., Lee Y.G., Kang K. (2015). Synergistic Multi-Doping Effects on the Li_7_La_3_Zr_2_O_12_ Solid Electrolyte for Fast Lithium Ion Conduction. Sci. Rep..

[35] Posch P., Lunghammer S., Berendts S., Ganschow S., Redhammer G.J., Wilkening A., Lerch M., Gadermaier B., Rettenwander D., Wilkening H.M.R. (2020). Ion Dynamics in Al-Stabilized Li_7_La_3_Zr_2_O_12_ Single Crystals–Macroscopic Transport and the Elementary Steps of Ion Hopping. Energy Storage Mater..

[36] Ihrig M., Mishra T.P., Scheld W.S., Häuschen G., Rheinheimer W., Bram M., Finsterbusch M., Guillon O. (2021). Li_7_La_3_Zr_2_O_12_ Solid Electrolyte Sintered by the Ultrafast High-Temperature Method. J. Eur. Ceram. Soc..

[37] Yamada H., Ito T., Hongahally Basappa R. (2016). Sintering Mechanisms of High-Performance Garnet-Type Solid Electrolyte Densified by Spark Plasma Sintering. Electrochim. Acta.

[38] Hong M., Dong Q., Xie H., Clifford B.C., Qian J., Wang X., Luo J., Hu L. (2021). Ultrafast Sintering of Solid-State Electrolytes with Volatile Fillers. ACS Energy Lett..

