# Supplementary material for: Synergetic Effect of Li-Ion Concentration and Triple Doping on Ionic Conductivity of Li7La3Zr2O12 Solid Electrolyte
Source: Nanomaterials (Basel). 2022 Aug 26;12(17):2946. doi: 10.3390/nano12172946 (PMC9457903; doi:10.3390/nano12172946)
Supplement: Supplementary file 1 [file nanomaterials-12-02946-s001.zip › nanomaterials-1875593-supplementary.pdf]

Supporting Information

Synergetic Effect of Li-Ion Concentration and Triple Doping on Ionic Conductivity of $Li_7La_3Zr_2O_{12}$ Solid Electrolyte

Minh Hai Nguyen and Sangbaek Park *

Department of Materials Science and Engineering, Chungnam National University, Daejeon 34134, Korea
* Correspondence: sb.park@cnu.ac.kr

Table S1. Average particle size of Al/Ga/Ta-doped LLZO powders prepared with different ball milling parameters through PSA measurement.

Sample	1st ball milling conditions	Calcination	2nd ball milling conditions	Average particle size (μm)
AGT_LLZO	250 rpm / 6 h	-	-	1.03
	250 rpm / 6 h	900 °C / 6 h	-	24.4
	250 rpm / 6 h	900 °C / 6 h	200 rpm / 2 h	1.66
	250 rpm / 6 h	900 °C / 6 h	300 rpm / 2 h	1.05
	250 rpm / 6 h	900 °C / 6 h	400 rpm / 2 h	1.12
	250 rpm / 6 h	900 °C / 6 h	500 rpm / 2 h	0.78

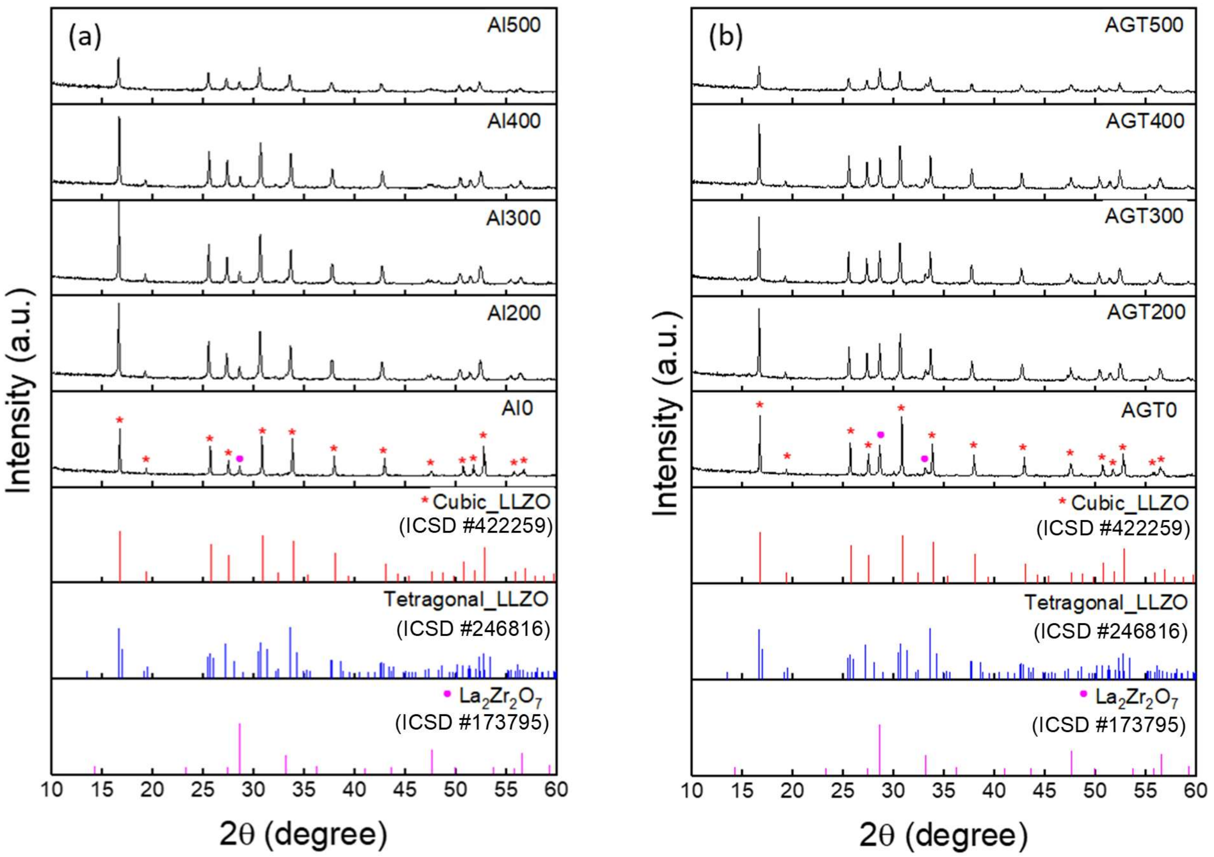

Figure S1: XRD patterns of (a) Al-doped LLZO powders and (b) Al/Ga/Ta-doped LLZO powders after the second ball milling process with different rotation speeds (0, 200, 300, 400, and 500 rpm).

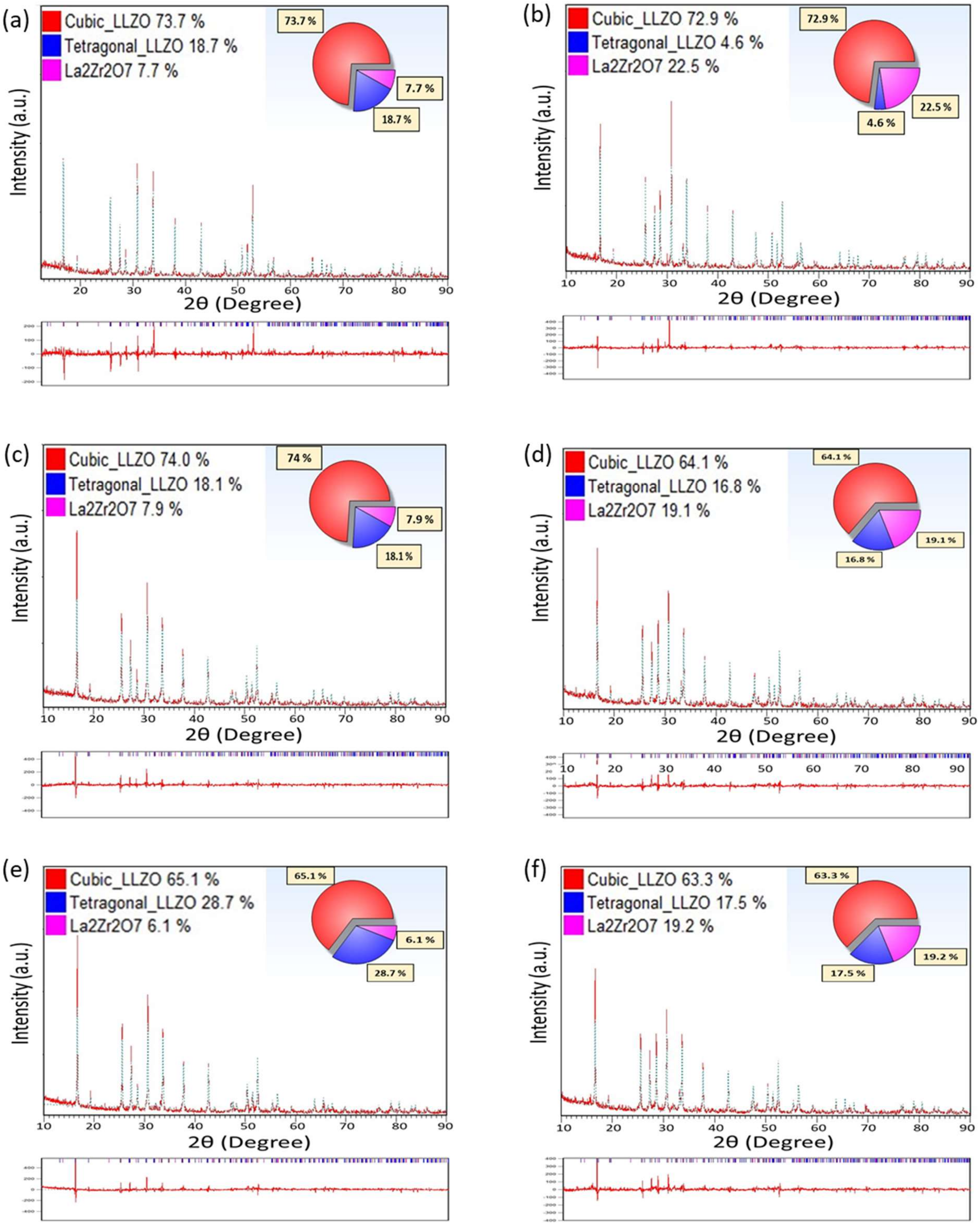
(a)
Cubic_LLZO 73.7 %
Tetragonal_LLZO 18.7 %
La2Zr2O7 7.7 %
73.7 %
7.7 %
18.7 %
Intensity (a.u.)
2θ (Degree)
(b)
Cubic_LLZO 72.9 %
Tetragonal_LLZO 4.6 %
La2Zr2O7 22.5 %
72.9 %
22.5 %
4.6 %
Intensity (a.u.)
2θ (Degree)
(c)
Cubic_LLZO 74.0 %
Tetragonal_LLZO 18.1 %
La2Zr2O7 7.9 %
74 %
7.9 %
18.1 %
Intensity (a.u.)
2θ (Degree)
(d)
Cubic_LLZO 64.1 %
Tetragonal_LLZO 16.8 %
La2Zr2O7 19.1 %
64.1 %
19.1 %
16.8 %
Intensity (a.u.)
2θ (Degree)
(e)
Cubic_LLZO 65.1 %
Tetragonal_LLZO 28.7 %
La2Zr2O7 6.1 %
65.1 %
6.1 %
28.7 %
Intensity (a.u.)
2θ (Degree)
(f)
Cubic_LLZO 63.3 %
Tetragonal_LLZO 17.5 %
La2Zr2O7 19.2 %
63.3 %
19.2 %
17.5 %
Intensity (a.u.)
2θ (Degree)

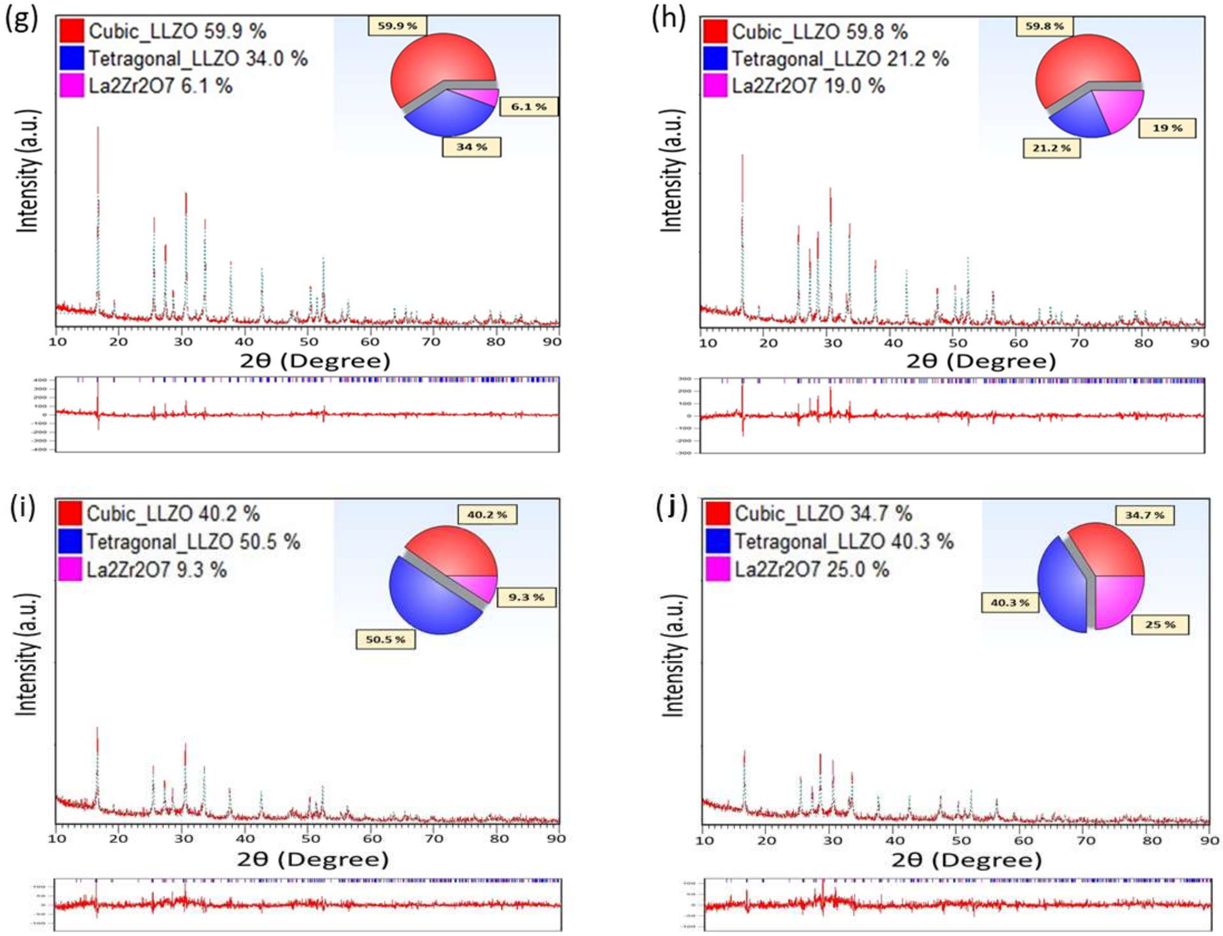

Figure S2: XRD Rietveld refinement results of Al-doped LLZO and AGT-doped LLZO powders prepared with Li content of 6.9 mol after the second ball milling process with different rotation speeds (0, 200, 300, 400 and 500 rpm): (a) Al0, (b) AGT0, (c) Al200, (d) AGT200, (e) Al300, (f) AGT300, (g) Al400, (h) AGT400, (i) Al500, and (j) AGT500.

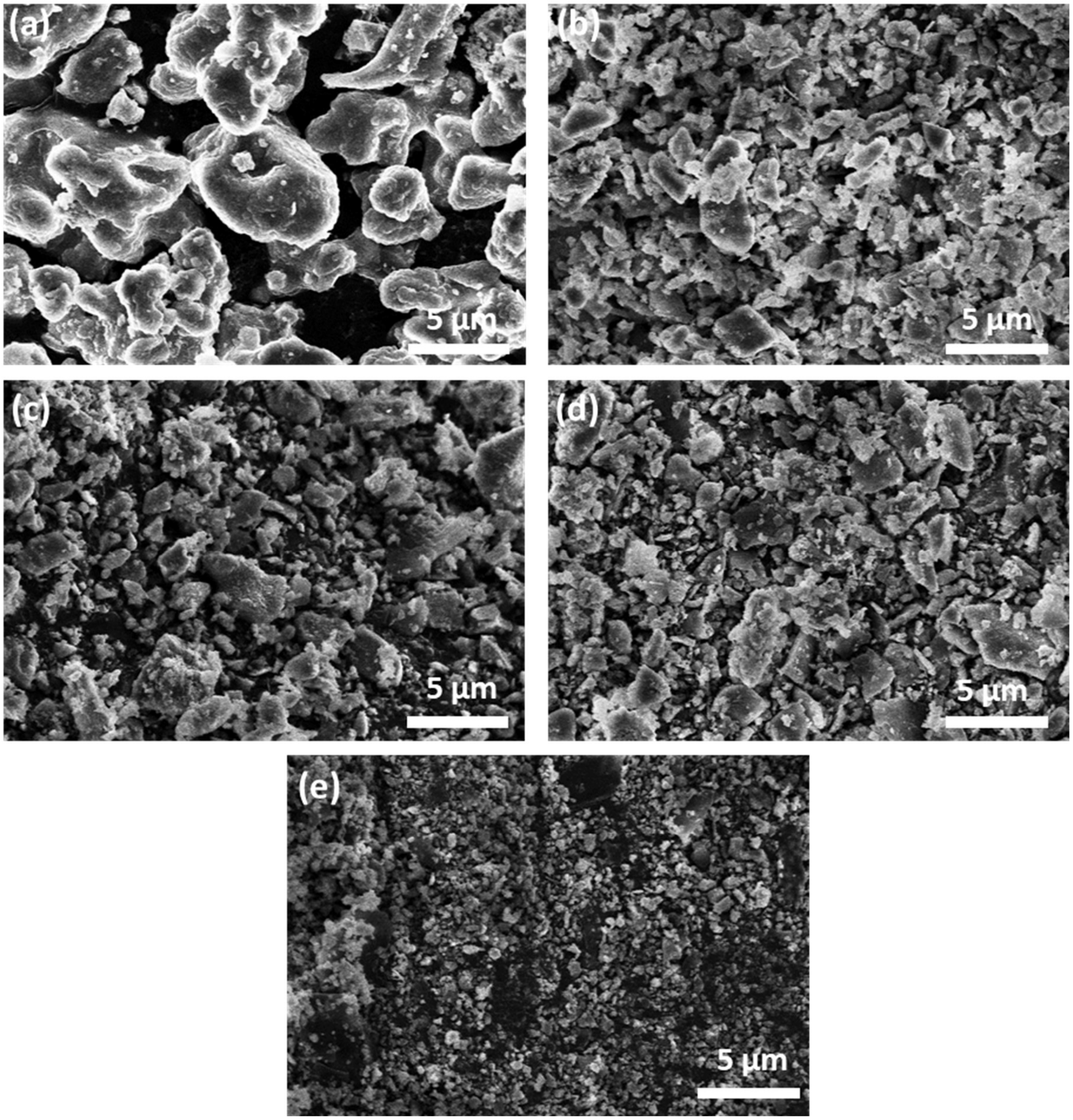

Figure S3: SEM images of the Al/Ga/Ta-doped LLZO powders after the second ball milling process at (**a**) 0 rpm, (**b**) 200 rpm, (**c**) 300 rpm, (**d**) 400 rpm, and (**e**) 500 rpm for 2 h.

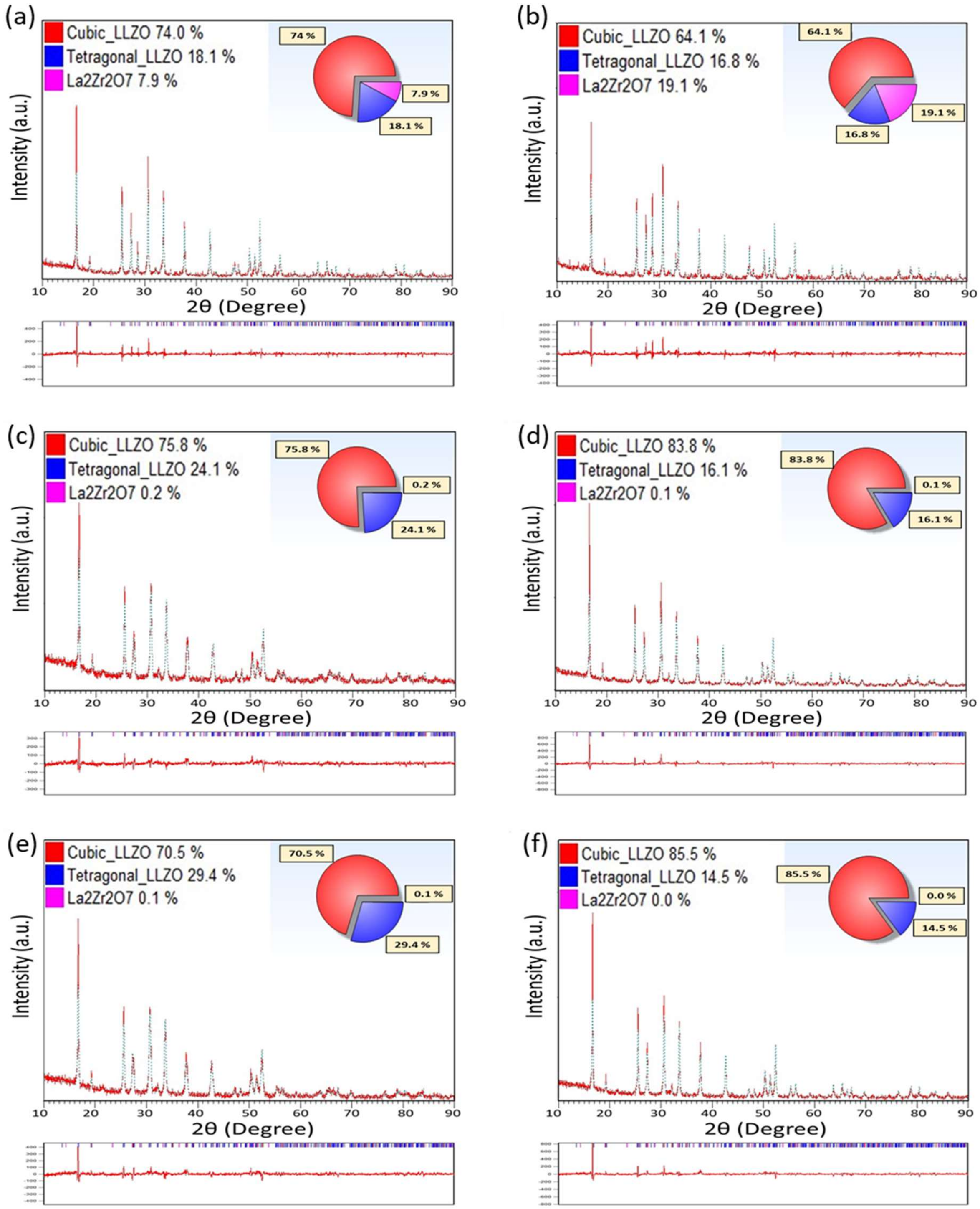

Figure S4: XRD Rietveld refinement results of powder samples after the second ball milling process at 200 rpm for 2 h: (**a**) Al_6.9, (**b**) AGT_6.9, (**c**) Al_7.7, (**d**) AGT_7.7, (**e**) Al_8.4, and (**f**) AGT_8.4.

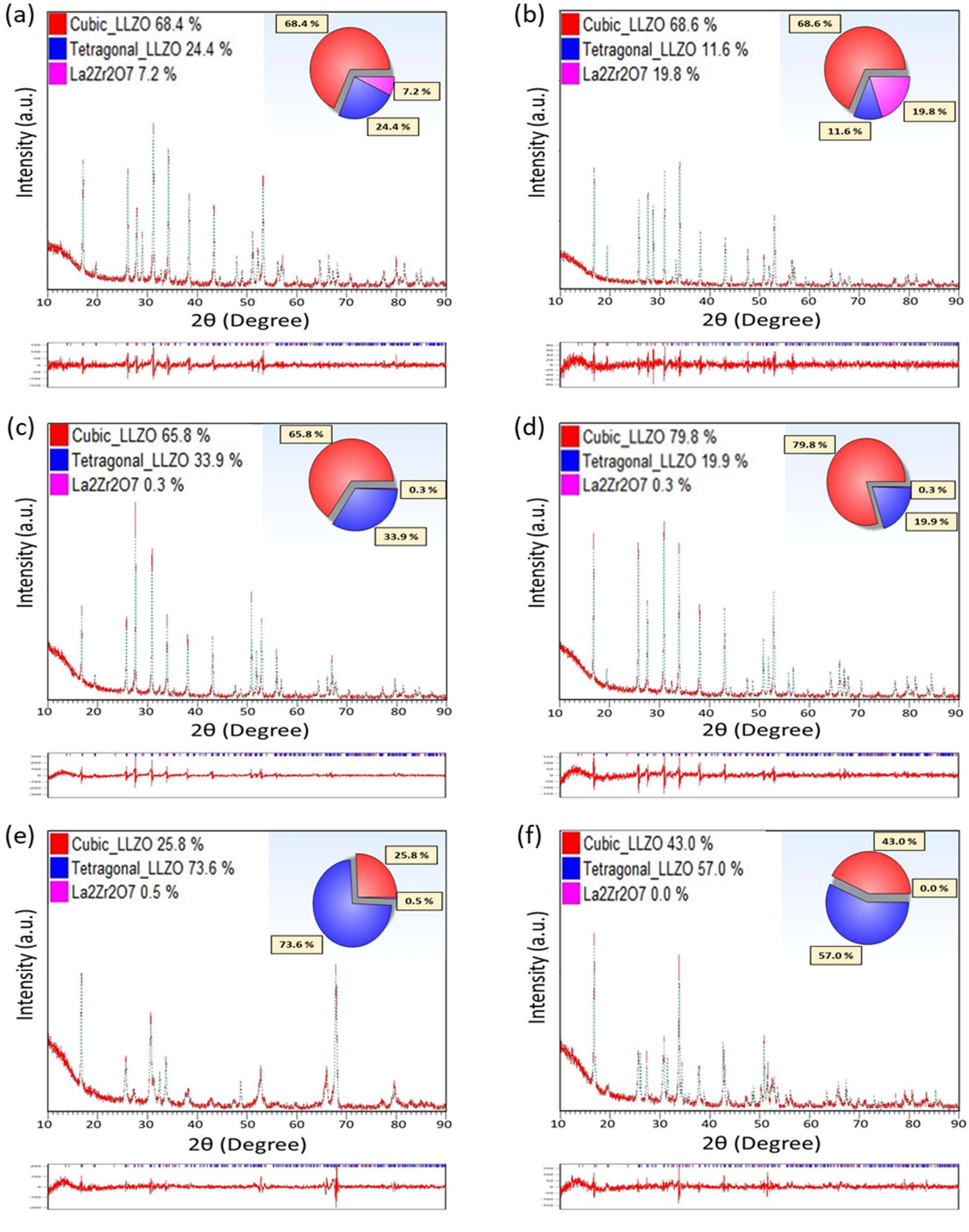

Figure S5: XRD Rietveld refinement results of pellets: (**a**) Al_6.9, (**b**) AGT_6.9, (**c**) Al_7.7, (**d**) AGT_7.7, (**e**) Al_8.4, and (**f**) AGT_8.4.

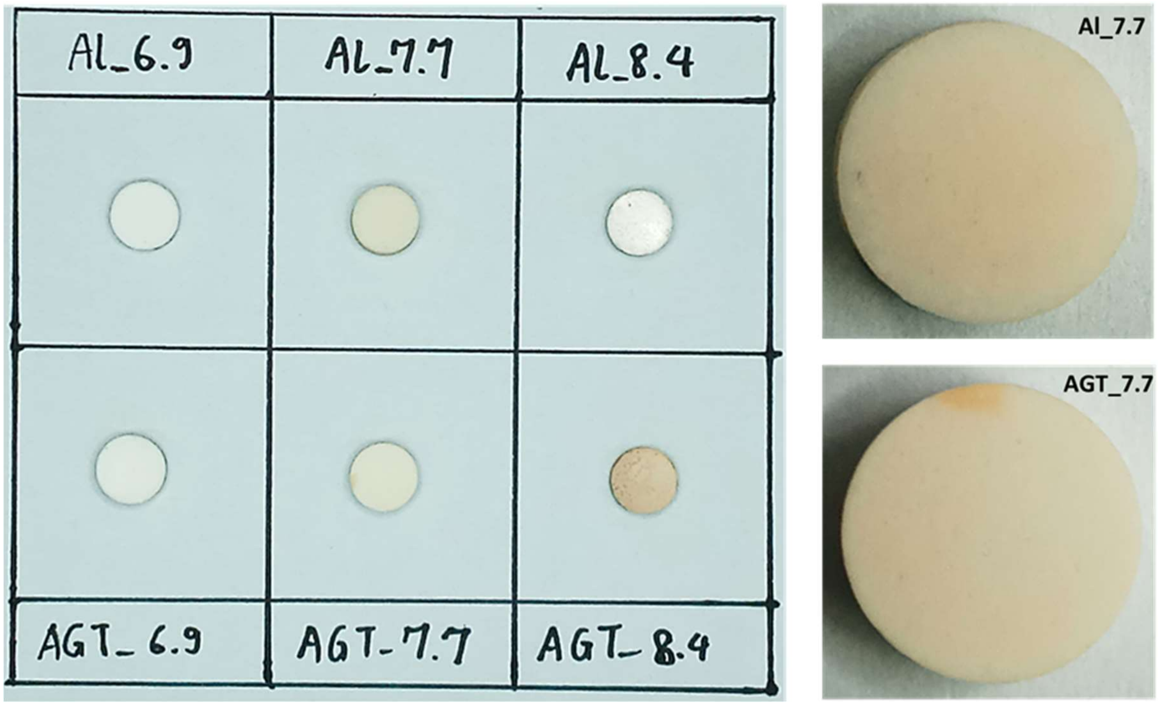

Figure S6: Digital image of pellets.

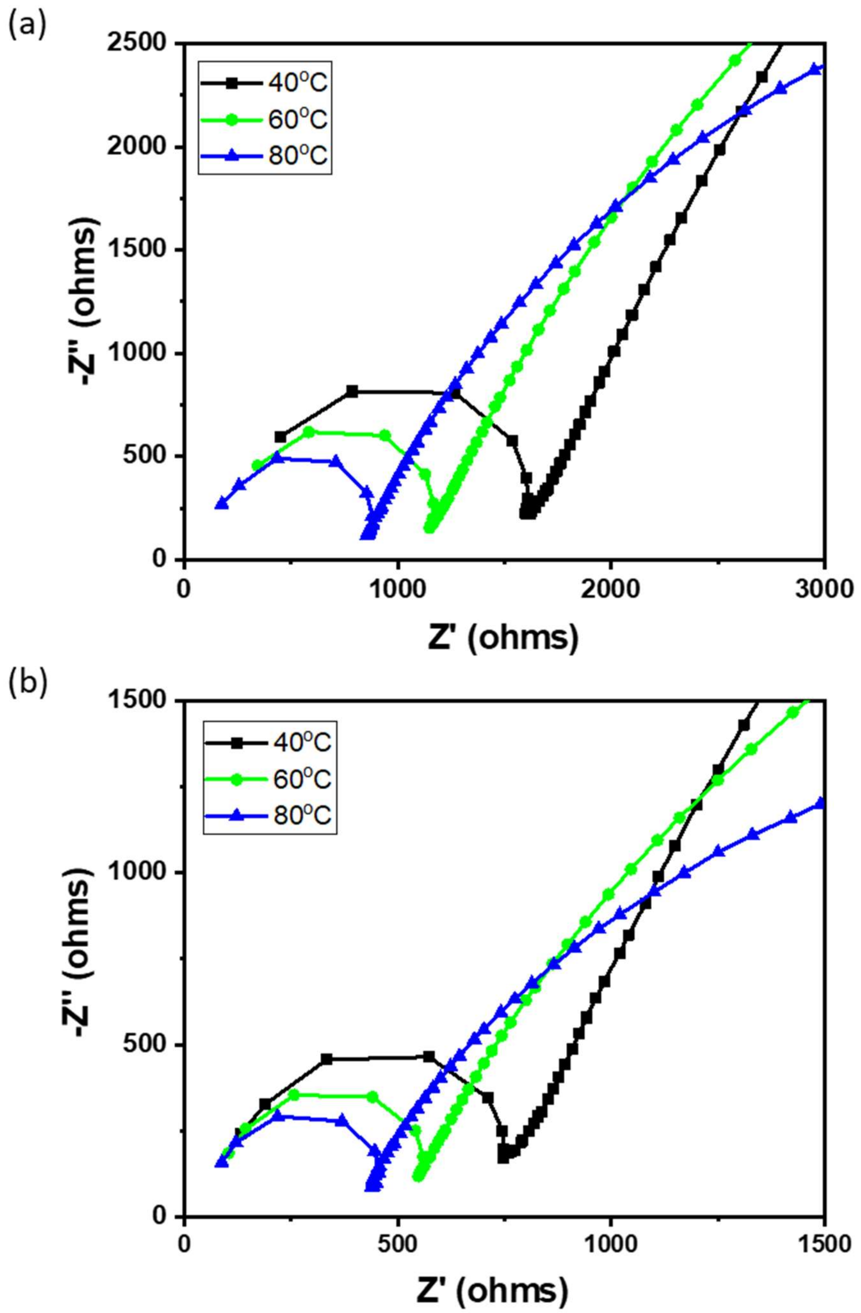

Figure S7: Nyquist plots of (**a**) Al_7.7 and (**b**) AGT_7.7 samples at 40–80 °C

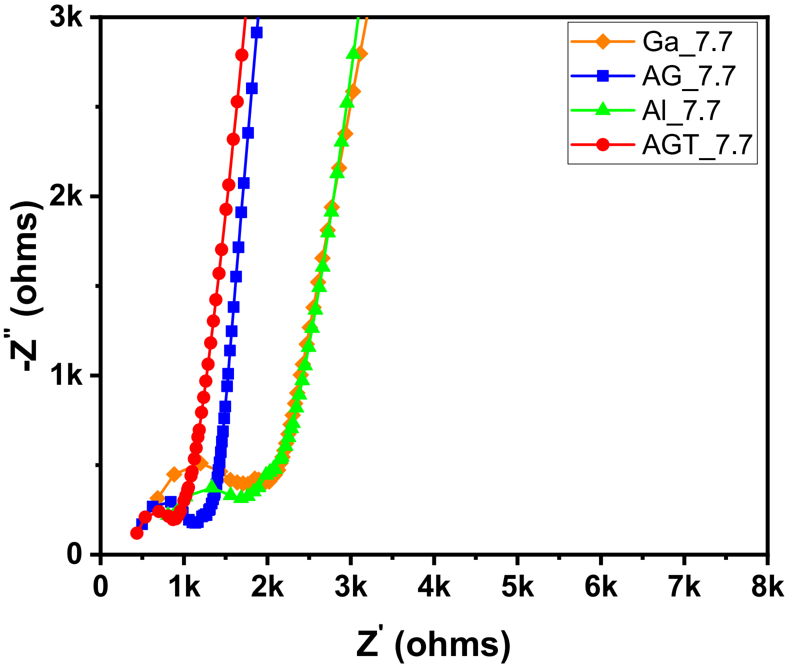

Figure S8: Nyquist plots of Al-doped LLZO, Ga-doped LLZO, Al/Ga-doped LLZO, and Al/Ga/Ta-doped LLZO pellets with Li^{+} concentration of 7.7 mol.

Table S2: Summary of total ionic conductivity at 25 °C of Al-doped LLZO, Ga-doped LLZO, Al/Ga-doped LLZO, and Al/Ga/Ta-doped LLZO pellets with Li^+ concentration of 7.7 mol.

**Sample**	**Al_7.7**	**Ga_7.7**	**AG_7.7**	**AGT_7.7**
Ion conductivity ($x10^{-4}$ S cm^{-1})	1.7	2.0	3.2	3.6

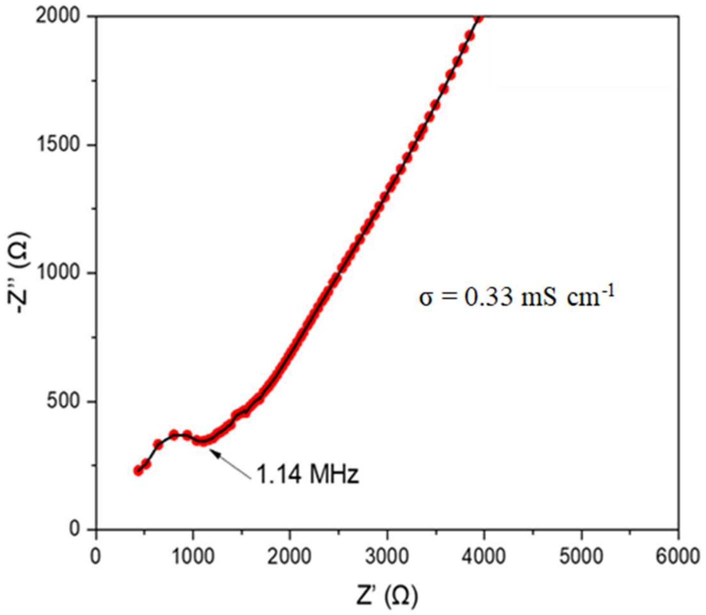

Figure S9: Nyquist plots of Al-doped LLZO prepared with Li^+ concentration of 7.7 mol and sintering time of 24 h.

Table S3: Comparison of LLZO pellets prepared by fast sintering methods in this work and other studies.

Composition	Sintering method	Sintering temperature (°C)	Sintering time	Ionic conductivity at 25 °C (mS cm^{-1})	Reference
$Li_{6.45}Al_{0.05}La_3Zr_{1.6}Ta_{0.4}O_{12}$	Ultrafast High-temperature Sintering (UHS) with an AC/DC power source	1500	10 s	0.12	[36]
$Li_{6.4}La_3Zr_{1.4}Ta_{0.6}O_{12}$	Furnace sintering (using Pt crucibles)	1250	40 min	0.64	[32]
$Li_{6.5}La_3Zr_{1.45}Ta_{0.55}O_{12}$	Sintering with DC power supply and volatile fillers	1327	20 s	1.09	[38]
$Li_{6.15}La_3Zr_{1.75}Ta_{0.25}Al_{0.2}O_{12}$	Hot pressing	1000	1 h	0.4	[31]
$Li_{6.15}La_3Zr_{1.75}Ta_{0.25}Al_{0.2}O_{12}$	Hot pressing	1050	1 h	0.37	[18]
$Li_{6.5}La_3Zr_{1.5}Ta_{0.5}O_{12}$	Spark plasma sintering	1000	10 min	0.69	[37]

$Li_{6.25}Al_{0.25}La_3Zr_2O_{12}$	Furnace sintering	1250	40 min	0.17	This work
$Li_{6.25}Al_{0.25}La_3Zr_2O_{12}$	Furnace sintering	1250	24 h	0.33	This work
$Li_{6.25}Al_{0.172}Ga_{0.072}La_3Zr_{1.982}Ta_{0.018}O_{12}$	Furnace sintering	1250	40 min	0.36	This work
